# Editorial: Spectroscopy, imaging and machine learning for crop stress

**DOI:** 10.3389/fpls.2023.1240738

**Published:** 2023-07-28

**Authors:** Shizhuang Weng

**Affiliations:** National Engineering Research Center for Agro-Ecological Big Data Analysis & Application, School of Electronic and Information Engnieering Anhui University, Hefei, China

**Keywords:** spectroscopy, machine vision, hyperspectral imaging, machine learning, deep learning, crop stress

Crop stress poses a huge challenge to food security necessitating innovative approaches for early detection monitoring management of stress. In recent years the integration of spectroscopy imaging machine learning techniques has emerged as a promising avenue for detecting various types of crop stress. This editorial work introduces recent publications within the field included in the Research Topic “*Spectroscopy imaging machine learning for crop stress*.” By exploring these cutting-edge research findings we can gain valuable insights into the application of these technologies to enhance agricultural resilience productivity.

Xiao et al. focused on the use of visible and near-infrared spectroscopy combined with deep learning to estimate leaf nitrogen concentration in cotton leaves. The study employed random frog, weighted partial least squares regression and saliency map to select characteristic wavelengths. The models based on convolutional neural network showed excellent performance for both qualitative and quantitative prediction of leaf nitrogen. These findings highlight the potential of deep learning and visible and near-infrared spectroscopy within accurate and real-time assessment of cotton leaf nitrogen content, enabling farmers to make applicable fertilization decisions

Wu et al. employed an unmanned aerial vehicle (UAV) to obtain multispectral images of a rice canopy and analyzed the response of multispectral reflectance features and physiological parameters including net photosynthetic rate (Pn), plant height (PH), and SPAD to different nitrogen treatments or leakage conditions at various growth stages of the crop. The study extensively analyzed the correlation between vegetation indices (VIs), texture indices (TIs), and Pn based on UAV multispectral images. The techniques and findings presented in this paper provide valuable insights within field-scale photosynthetic monitoring in rice cultivation and improve stress detection and yield prediction.

Choudhury et al. introduces two innovative algorithms, namely VisStressPredic and HyperStressPropagateNet, to predict the onset and propagation of drought stress in plants using camera-captured image sequences in visible light and hyperspectral modalities. The algorithms analyze visible light sequences at discrete intervals and utilize a deep neural network and hyperspectral images to propagate stress over time, which demonstrate a strong correlation between soil water content and the percentage of stressed plants. These algorithms are evaluated on a dataset of cotton plant image sequences and offer valuable support for studying abiotic stresses in diverse plant species, promoting sustainable agricultural practices.

Hu X. et al. propose a novel approach called Class-Attention-based Lesion Proposal Convolutional Neural Network (CALP-CNN), utilizing a class response map to locate the main lesion object and identify discriminative lesion details in visual light images. Through a cascade architecture, CALP-CNN effectively handles complex background interference and misclassification of similar diseases. Experimental results on a self-built dataset demonstrate CALP-CNN achieves good classification performance and outperforms the existing fine-grained image recognition methods, highlighting its efficacy in field identification of strawberry diseases.

Hu Y. et al. presents an enhanced encoder-decoder framework based on DeepLab v3+ analysis of images to accurately identify crops with diverse planting patterns. The network utilizes ShuffleNet v2 as the backbone for feature extraction and incorporates a convolutional block attention mechanism to fuse attention features across channels and spatial dimensions. The enhanced network achieves significant improvements and requires fewer parameters and computational operations compared to other networks. This study demonstrates the effectiveness of Deep-agriNet in identifying crops with different planting scales, making it a valuable tool for crop identification in various regions and countries.

The combination of spectroscopy, imaging, and machine learning has a high potential for improving crop stress analysis and management. By utilizing these technologies, we can enhance our understanding of crop stress dynamics, develop precise and targeted stress detection methods, and improve decision-making processes for farmers. Ongoing research, technological advancements, and collaborative efforts are necessary to unlock the full potential of spectroscopy, imaging, and machine learning in mitigating crop stress and ensuring global food security.

## Author contributions

The author confirms being the sole contributor of this work and has approved it for publication.

